# Unraveling aromaticity: the dual worlds of pyrazole, pyrazoline, and 3D carborane

**DOI:** 10.3762/bjoc.21.29

**Published:** 2025-02-21

**Authors:** Zahra Noori, Miquel Solà, Clara Viñas, Francesc Teixidor, Jordi Poater

**Affiliations:** 1 Departament de Química Inorgànica i Orgànica & Institut de Química Teòrica i Computacional (IQTCUB), Universitat de Barcelona, Martí i Franquès 1-11, 08028 Barcelona, Spain; 2 Institut de Química Computacional i Catàlisi and Departament de Química, Universitat de Girona, Maria Aurèlia Capmany 69, 17003 Girona, Catalonia, Spain; 3 Institut de Ciència de Materials de Barcelona, Consejo Superior de Investigaciones Científicas, Campus Universitat Autònoma de Barcelona, 08193 Bellaterra, Spain; 4 ICREA, Pg. Lluís Companys 23, 08010 Barcelona, Spain

**Keywords:** 3D aromaticity, carborane, density functional theory, NICS ambiguity, pyrazole

## Abstract

A new series of *o*-carborane-fused pyrazoles has been recently successfully synthesized. This fusion was expected to create a hybrid 3D/2D aromatic system, combining the 3D aromaticity of *o*-carborane with the 2D aromaticity of pyrazole. However, while the boron cage retains its aromatic character, the pyrazole’s aromaticity is lost. As a result, rather than forming *o*-carborane-fused pyrazoles, the synthesis yielded *o*-carborane-fused pyrazolines, which are non-aromatic. The limited overlap between the π molecular orbitals (MOs) of the planar heterocycle and the *n* + 1 MOs of the carborane prevents significant electronic delocalization between the two fused components. This contrasts with the fusion of pyrazole and benzene to form indazole, where both rings maintain their 2D aromaticity. Our findings demonstrate that the peripheral σ-aromaticity of carborane and the π-aromaticity of the heterocycle are orthogonal, making a true 3D/2D aromatic system unachievable. The carborane is highly aromatic, generating highly negative NICS values (−25 to −30 ppm). We have observed that these high NICS values extend to fused rings, leading to incorrect estimations of aromaticity. Therefore, relying solely on NICS can be misleading, and other computational indicators, along with experimental or structural data, should be used to accurately assess aromaticity.

## Introduction

Pyrazoles and 1,2-diazoles are five-membered aromatic heterocyclic compounds that have garnered significant attention in recent years [1–3]. While these compounds are rarely found in nature, they exhibit a wide range of biological activities, making them highly useful in pharmaceutical chemistry [4–6]. Pyrazoles are also extensively employed in agrochemicals, serving as key components in insecticides, herbicides, and fungicides [7]. Beyond their chemical uses, pyrazoles play an important role in the construction of supramolecular assemblies and molecular systems designed for photoinduced electron transfer [8–9]. Thanks to their notable photophysical properties, pyrazoles are applied in OLED technology [10]. Noticeably, in its ground state, pyrazole (C_3_H_4_N_2_) is an aromatic molecule that follows Hückel's rule, with two formal double bonds and a lone pair on one nitrogen generating a π system with 6 π electrons [11]. When fused with a benzene ring, sharing a C–C bond, it remains aromatic, which is the case of indazole. Pyrazoline (C_3_H_6_N_2_), similar to pyrazole, formally has only one double bond and a lone pair on the nitrogen, so it does not satisfy Hückel's rule and it is therefore non-aromatic. Even when fused with benzene via a C–C bond, pyrazoline remains non-aromatic, which is the case of indazoline.

Icosahedral carboranes are globular molecular clusters made of carbon and boron, displaying 3D aromaticity [12–15]. Their unique properties – such as aromaticity, exceptional thermal and chemical stability, and robust synthetic versatility [16–17] – make carborane derivatives essential components in various fields. These include pharmaceuticals [18–22], boron neutron capture therapy (BNCT) [23–26], organometallic ligands [27], and functional materials [28–30]. As a result, developing efficient methods for selectively introducing functional groups into carboranes has become a key area of research [29,31]. Moreover, replacing planar aryl rings in biologically active molecules with spherical carborane units has led to novel alternatives [32], offering enhanced properties and efficacy [28,30,33–35]. Therefore, the advancement of simple, efficient methodologies for synthesizing *o*-carborane-fused heterocycles is critically needed. However, the limited number of synthetic strategies for carborane functionalization [36] continues to constrain their broader use.

With this in mind, Lee and colleagues have recently developed an efficient synthetic method for producing *o-*carborane-fused pyrazoles as a novel scaffold, without using transition metals. Their approach involves reacting B(4)-acylmethyl and B(3,5)-diacylmethyl *o*-carborane with 2-azido-1,3-dimethylimidazolinium hexafluorophosphate (ADMP) in the presence of DBU in acetonitrile. This one-pot process enables sequential diazotization and cyclization, leading to the formation of two or three C–N bonds under extremely mild conditions, with excellent tolerance for various functional groups [37]. A priori, this fusion between 3D aromatic *o*-carborane and 2D aromatic pyrazole should give a 3D/2D aromatic *o*-carborane-fused pyrazole. However, we recently demonstrated that, unlike many 2D/2D and 3D/3D aromatic fusions that retain their aromaticity, a 3D/2D aromatic combination is not feasible due to the ineffective overlap between the π molecular orbitals of the planar species and the (*n* + 1) molecular orbitals of the aromatic cage. This lack of overlap prevents effective electronic delocalization between the two fused units [38]. Soon after, Kelemen and colleagues confirmed our findings also applied to *o*-carboranes fused with five-membered ring systems [39–40]. The positioning of the heteroatom in these exo rings governs bonding, leading to restricted conjugation and, consequently, no aromatic stabilization. Importantly, the magnetic field generated by the 3D cluster influences the conjugation and the computed magnetic properties of the fused exo ring [34,38,40], which can lead to the incorrect assignment of aromatic character to this ring.

This study aims to determine whether *o*-carborane-fused pyrazoles can be classified as aromatic 3D/2D systems. While previous research has not specifically examined pyrazoles in this context, we hypothesize that although the 3D *o*-carborane maintains its aromaticity, the 2D pyrazole may lose its aromatic character. If our hypothesis holds, these compounds should be termed *o*-carborane-fused pyrazolines rather than pyrazoles. Alternatively, the presence of the N–N bond in pyrazole might help preserve its aromaticity. To explore this, we performed quantum chemical analyses on a range of *o*-carborane-fused pyrazoles and pyrazolines and compared them with their fully planar indazole and indazoline analogues. Aromaticity or non-aromaticity can be assessed using indicators such as magnetic-based NICS, electronic-based MCI, or bond lengths, among others, given its multidimensional character [41–43].

## Results and Discussion

**Figure 1:**
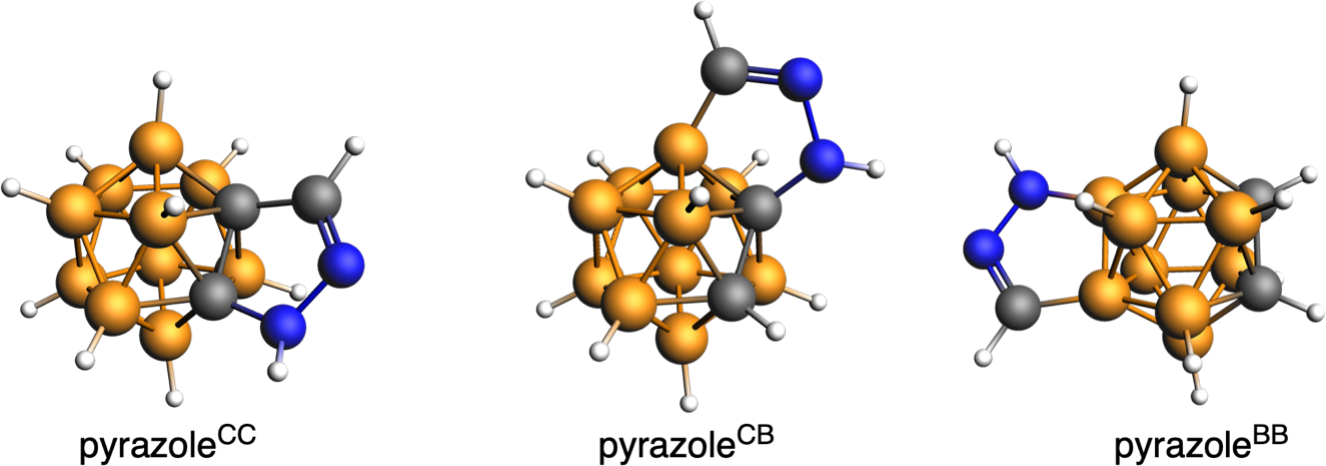
Series *o*-carborane-fused pyrazoles under analysis.

**Figure 2:**
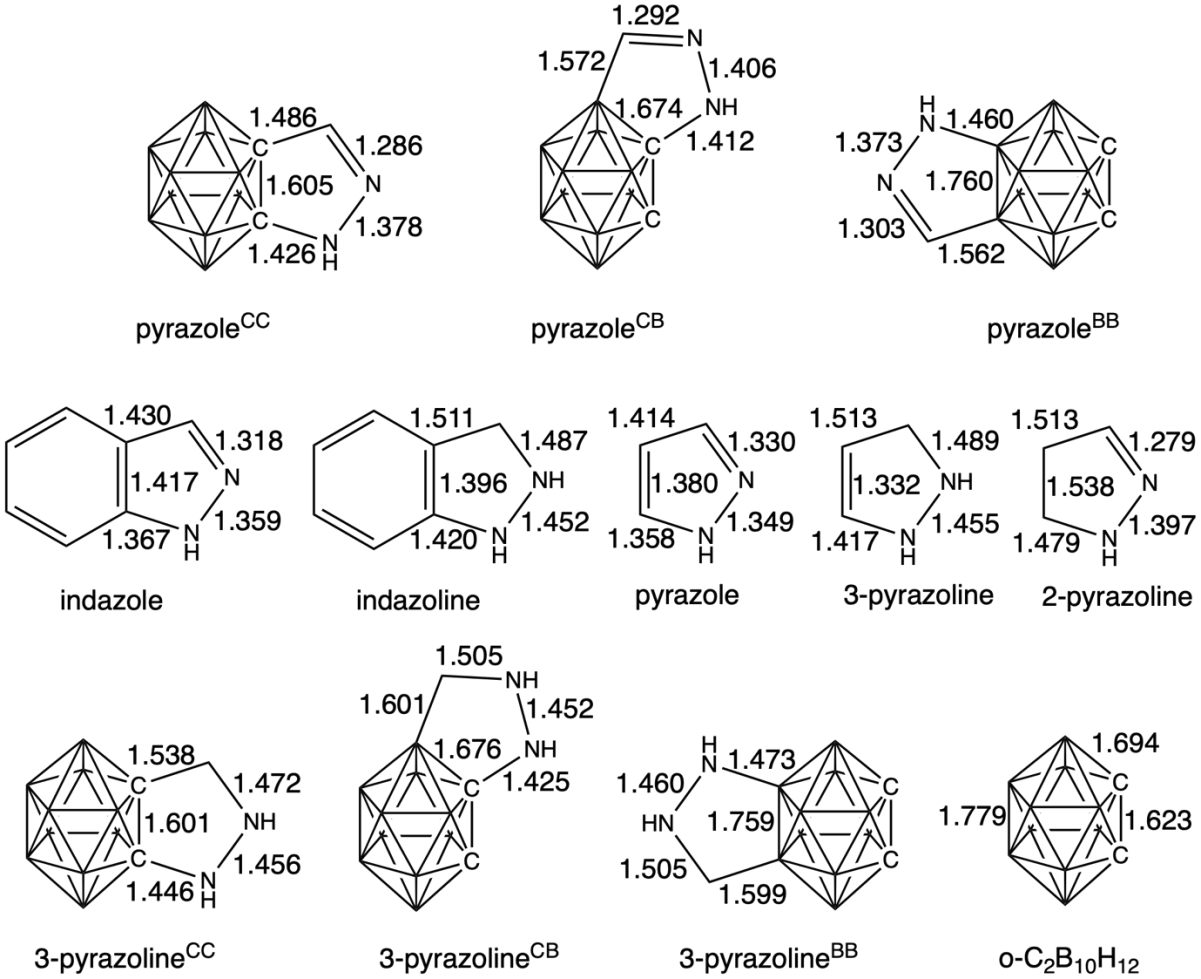
Bond lengths (in Å) of systems under analysis (top row) and reference systems (second and third rows) from the fusion of *o*-carborane and pyrazole/pyrazoline.

We have first analyzed a series *o*-carborane-fused pyrazoles involving the fusion to either a C–C, C–B or B–B bond in the boron cluster, referred as pyrazole^CC^, pyrazole^CB^, and pyrazole^BB^, respectively (Figure 1). Among these isomers, pyrazole^BB^ is the most stable, followed by pyrazole^CB^ and pyrazole^CC^ by 19.2 and 24.5 kcal mol^−1^, respectively. This order of stability correlates with the length of the fusing bond between the carborane and the pyrazole, i.e., B–B, C–B and C–C decrease from 1.760, to 1.674 and to 1.605 Å, respectively (Figure 2). Thus, the longer the length of the fusing bond, the lower the tension of the formed five-membered ring and the more stable the complex. The stronger strength of the C–B bond compared to the C–C bond by 2.4 kcal/mol further supports the above statement [44].

**Figure 3:**
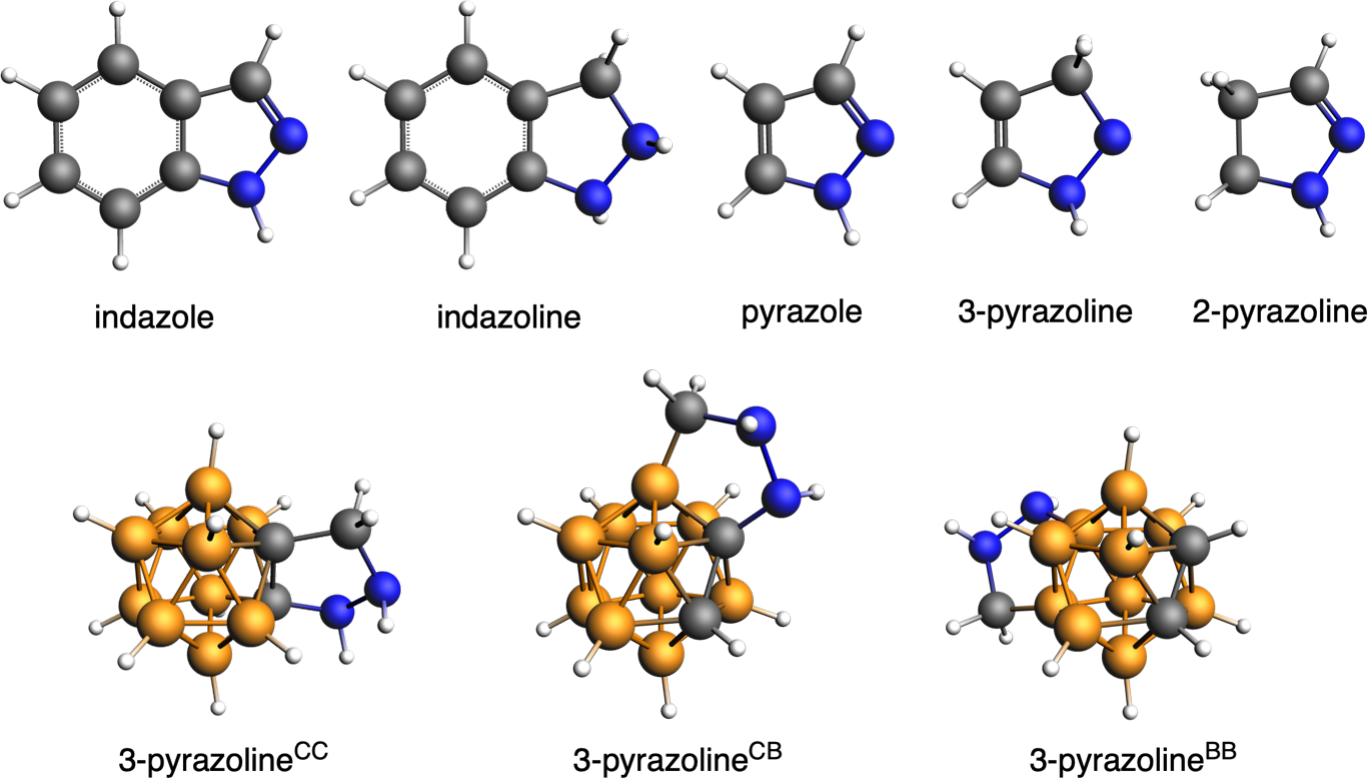
Series of reference systems for the *o*-carborane-fused pyrazoles under analysis.

Next, we have compared the above *o*-carborane-fused pyrazoles with a series of reference systems with the aim to better understand the electronic structure of the fused heterocycle and its aromaticity (Figure 3). First, the C–C bond length connecting the boron cage and pyrazole is 1.605 Å (pyrazole^CC^), very similar to that of *closo*-C_2_B_10_H_12_ [45–46] computed at the same level of theory (1.623 Å), a length that should be assigned to less than a single C–C bond as it shall be because the lines represent connections and not bonds (Figure 2). At difference, that of pyrazole is 1.380 Å, i.e., a bond length characteristic of a double C–C bond. Or even more, if instead of fusing the pyrazole to the *o*-carborane, we fuse it to benzene, and thus we have indazole, the fusing C–C bond length amounts to 1.417 Å, that can also be assigned to an aromatic C–C bond (that of benzene is 1.394 Å, computed at the same level of theory). Noticeably, highly diagnostic are also the NN bond lengths. In the aromatic indazole and pyrazole this NN distance is near 1.35 Å, whereas it is longer near 1.46 Å for the non-aromatic 3-pyrazoline, although 2-pyrazoline presents a shorter 1.40 Å distance (Figure 2). Thus, and based on the above geometrical data, compared to either pyrazole or indazole molecules, can the pyrazole fused to *o*-carborane be referred as pyrazole? Or otherwise, should we refer to this five-membered ring as pyrazoline due to the fact that the fusing C–C bond between *o*-carborane and the pyrazole is not a double bond? Finally, in agreement with the above discussion on the bond lengths, Pierrefixe and Bickelhaupt revealed the underlying electronic bonding mechanisms in the π-electron and σ-electron systems that cause the typical aromatic bond-length patterns in aromatic and heteroaromatic species. In particular, the authors proved the propensity of the π electrons to localize double bonds against the delocalizing force of the σ electrons [47–49].

**Figure 4:**
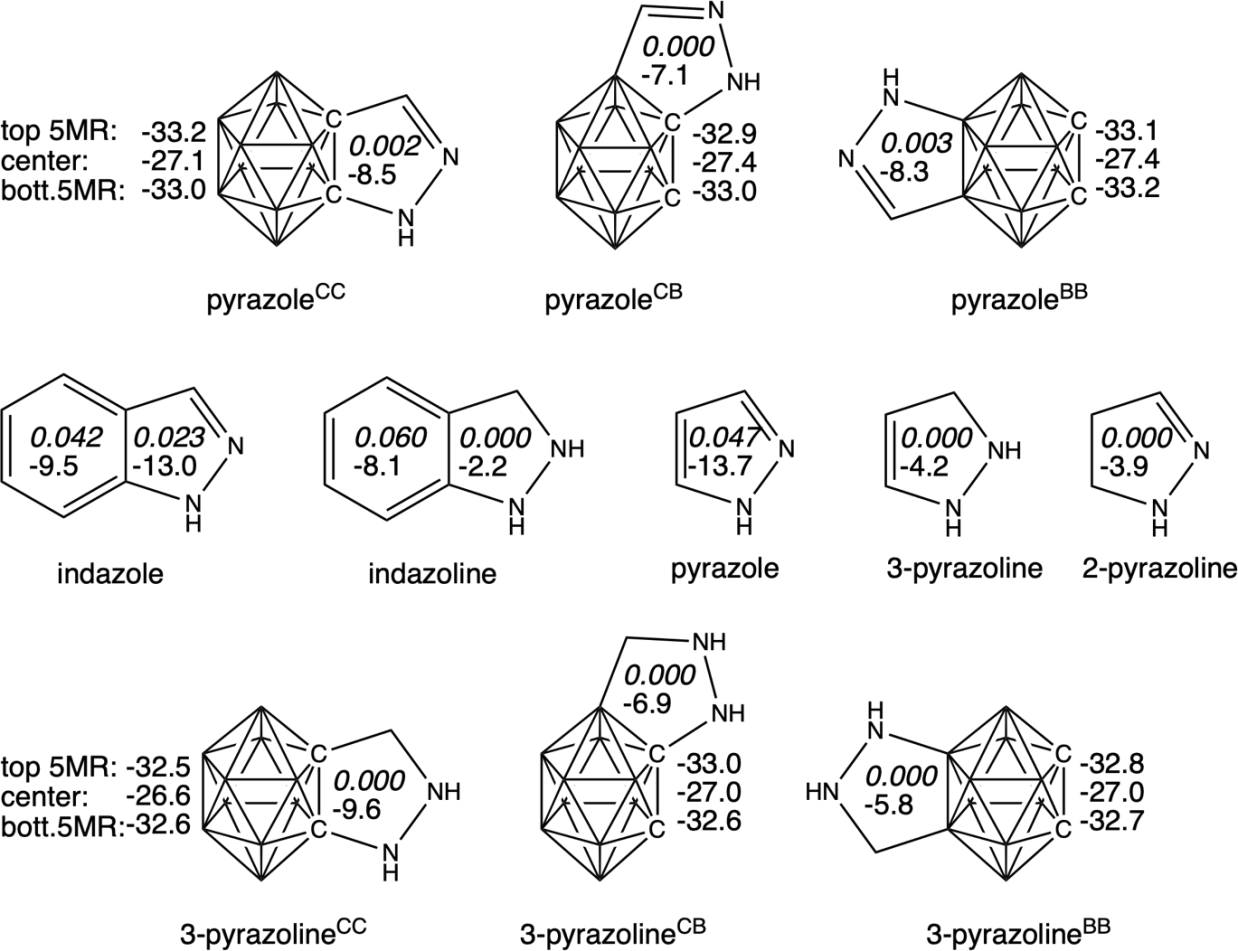
NICS (in ppm) of the boron cages (computed for the top 5-membered ring, center and bottom 5-membered ring) and the rings (center of the ring) of the systems under analysis from the fusion of *o*-carborane and pyrazole/pyrazoline and reference systems. MCI for indazole/pyrazole are also included (top value in italics).

Let us focus now on the aromaticity of the *o*-carborane-fused pyrazoles under analysis (Figure 4). The aromaticity of the *o*-carborane is hardly affected by the fusion to the pyrazole, with NICS in the center of the cage that amount to −27.1, −27.4, and −27.4 ppm for pyrazole^CC^, pyrazole^CB^, and pyrazole^BB^, respectively (compared to −27.3 ppm for *closo*-C_2_B_10_H_12_). However, whereas the boron cage is aromatic, the heterocyclic ring is clearly non-aromatic, with MCI that amount to 0.002, 0.000 and 0.003 a.u., respectively (Figure 4). At difference, based on NICS, these heterocycles should be considered aromatic (−8.5, −7.1, and −8.3 ppm, respectively). However, we have previously proven that this abnormal NICS values are caused by the induced magnetic ring current of the *o*-carborane [15,34,39]. This suggests we should exercise caution when assessing the aromaticity of a ring based solely on magnetic criteria such as NICS [50–53], especially in cases where neighboring cycles exhibit high or very high NICS values. For a more reliable evaluation, it is important to also consider structural factors and additional indicators like the multicenter index (MCI) [54–57]. Thus, from now on, we will evaluate the aromaticity of heterocycles solely using the electron-based MCI criterion [58–59].

**Figure 5:**
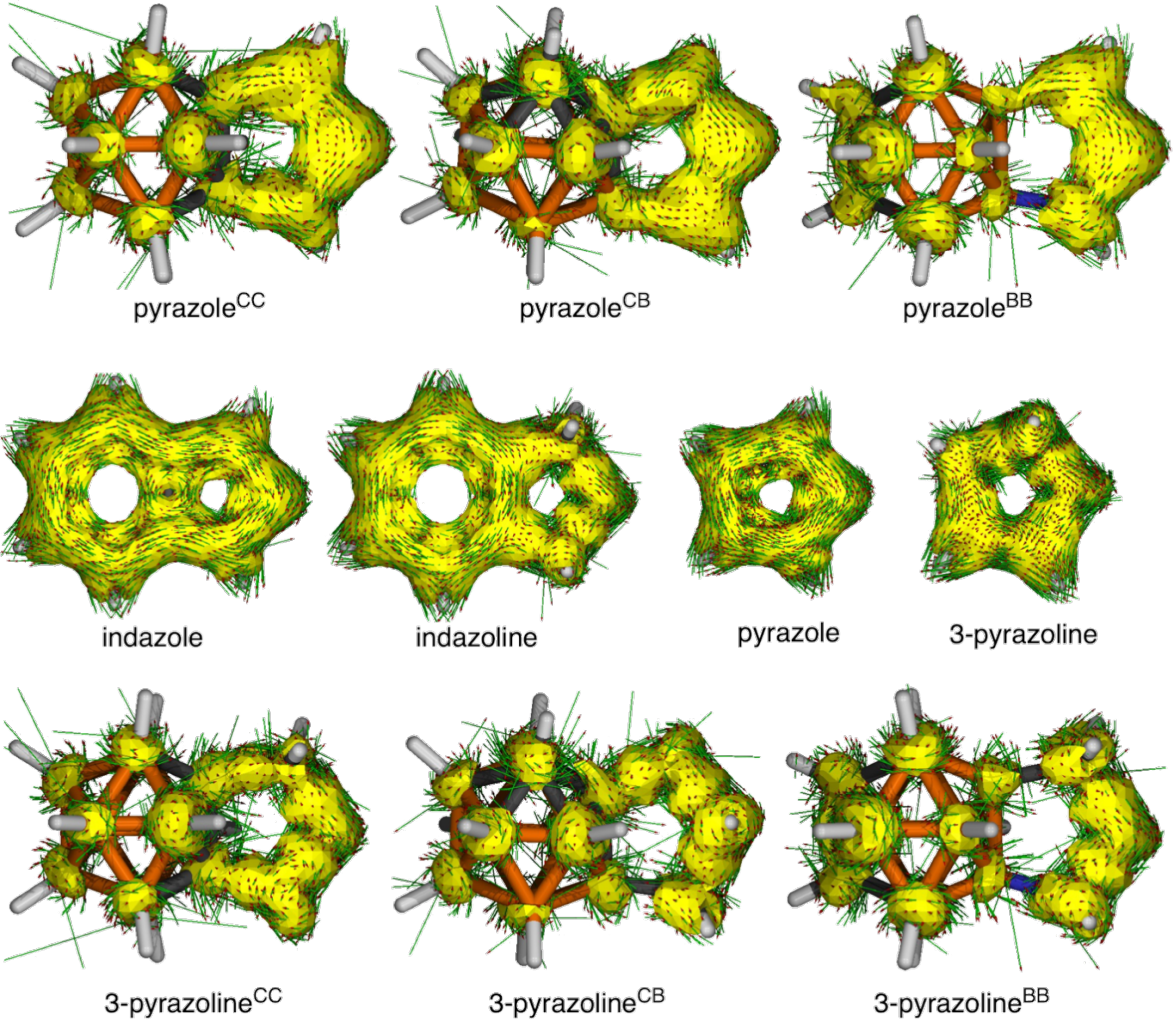
AICD plots of systems under analysis from the fusion of *o*-carborane and pyrazole/pyrazoline and reference systems (isosurface = 0.04).

**Figure 6:**
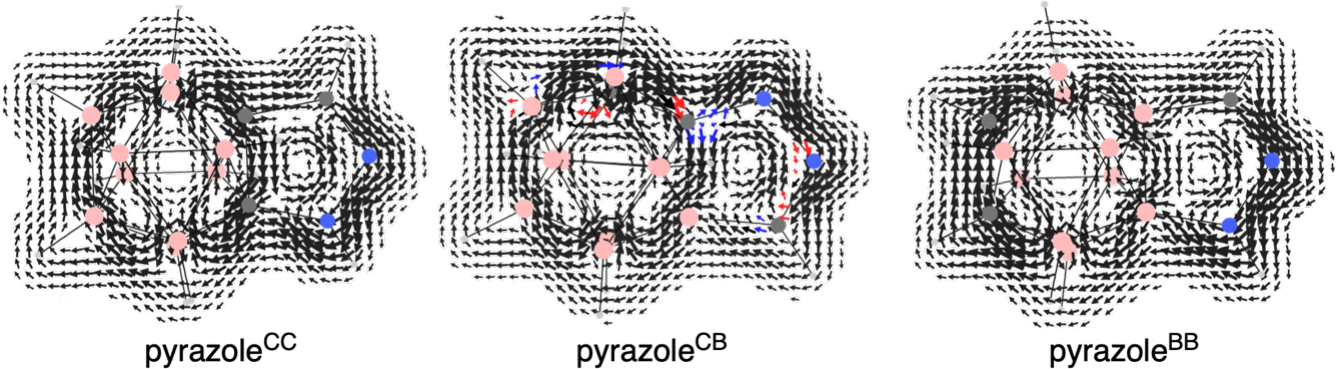
Current density maps (all-electron contributions) for a perpendicular magnetic field over a plane 1 a.u. above the molecular plane of the pyrazole ring of the *o*-carborane-fused pyrazoles. Red/blue arrows when the component parallel/antiparallel to **B** is greater than 30% of the vector modulus. Diatropic/paratropic circulations are clockwise/anticlockwise. Figure S3 in Supporting Information File 1 encloses the rest of the systems.

At difference to the *o*-carborane-fused pyrazoles, the pyrazole molecule is clearly aromatic with MCI = 0.047 a.u. (MCI for benzene is 0.072 a.u., computed at the same level of theory), whereas the aromaticity of the five-membered ring of indazole is reduced to 0.023 a.u. The aromaticity of both indazole and pyrazole molecules is further supported by the computed AICD plots (Figure 5), clearly showing a strong diatropic ring current around the five-membered rings. At difference, such current is interrupted in case of all *o*-carborane-fused pyrazoles between the cage and the heterocycle. This latter conclusion is further supported by the computed current density maps for the *o*-carborane-fused pyrazoles (Figure 6). It is observed a non-continuous diatropic ring current in the five-membered ring, interrupted by the fusing bond between the cage and the pyrazole with a paratropic current. Meanwhile, the cage is confirmed to be 3D aromatic with diatropic ring currents that extent to the five-membered ring. Once again, if the pyrazole ring fused to *o*-carborane is confirmed to be non-aromatic, can we still refer to it as pyrazole?

At this stage, it is important to reference a recent study in which we sought to gain a deeper understanding of the C–C bond in *o*-carborane by comparing *o*-carboryne and *o*-benzyne [60]. Noticeably, we found out that although *o*-carboryne and *o*-benzyne share similarities, the nature of the C–C bond formed between two adjacent carbons following the loss of hydrogen atoms differs. In *o*-benzyne, the C–C bond behaves as a triple bond, while in *o*-carboryne, it is a double bond.

Thus, in the present case with pyrazole, once again the ineffective overlap between the π molecular orbitals of the planar pyrazole and the (*n* + 1) molecular orbitals of the aromatic cage causes the aromaticity of the former to be vanished. Such ineffective overlap has been proven by means of a model system derived from pyrazole^CC^ in which the C–N and C–C bonds linking the carborane to the pyrazole have been broken and its interaction has been analyzed by means of a Kohn–Sham molecular orbital analysis together with an energy decomposition analysis (Figure S1 in Supporting Information File 1). To form the broken bonds, both fragments have two unpaired electrons (at their triplet state). The interaction between both fragments amounts to −227.9 kcal mol^−1^, mainly driven by the very attractive orbital interaction (∆*E*_oi_ = −543.7 kcal mol^−1^) due to the favorable interaction between the two single-occupied molecular orbitals (SOMO) of each fragment to form the two broken bonds. Such strong interaction is supported by large overlaps between these SOMO of each fragment (Table S1 in Supporting Information File 1). However, the interaction between the π molecular orbitals of the pyrazole fragment (HOMO−1 and HOMO−2, Figure S2 in Supporting Information File 1) and those of the carborane is very weak, as supported by the small overlaps between these orbitals.

On the other hand, the fact that the fusing C–C bond between the *o*-carborane and the pyrazole is not a double bond is the reason why we question referring to the five-membered ring as pyrazole. Instead, this ring should be referred as pyrazoline. For this reason, we have also analyzed the fusion between *o*-carborane and pyrazoline (Figure 3). In particular, if we assume that the fusing C–C linkage is single, the fused five-membered ring corresponds to 2-pyrazoline. This latter, as a molecule alone is clearly non-aromatic (MCI = 0.000). For comparison, we have also considered the fusion of *o*-carborane to 3-pyrazoline (13.0 kcal mol^−1^ higher in energy). Also in this case, the five-membered rings are clearly confirmed to be non-aromatic (Figure 4).

## Conclusion

In this work, we have quantum chemically analyzed a series of *o*-carborane-fused pyrazoles that have been recently synthesized, and whose fusion was expected to create a hybrid 3D/2D aromatic system, combining the 3D aromaticity of *o*-carborane with the 2D aromaticity of pyrazole. Notably for the case of pyrazole or pyrazoline, the N–N bond length is diagnostic, being approximately 0.1 Å longer when the molecule lacks aromaticity. In contrast, *o*-carborane (1,2-C_2_B_10_H_12_) is 3D aromatic and follows Wade–Mingos’ rule [61–63]. When fused to a C_3_N_2_ five-membered ring, that could lead to a pyrazole or pyrazoline moiety, the cluster retains its aromaticity, but the C_3_N_2_ five-membered ring does not. Our interpretation, consistent with our previous results, suggests that this phenomenon arises because carborane exhibits peripheral σ-aromaticity, while pyrazole shows π-aromaticity, and these two types of bonding are orthogonal.

For pyrazole to maintain aromaticity, either the C–C, C–B, or B–B bond in the carborane/pyrazole-fusing linkage would need to exhibit double-bond character. However, these bonds are weaker than a typical single bond. Thus, it is difficult to preserve global aromaticity when combining a 3D aromatic system with a 2D one. The MCI indicator, along with the C–C, C–N, and N–N bond distances – particularly the latter – suggest that the fused pyrazole ring is more accurately described as pyrazoline.

It is important to note that NICS values might incorrectly indicate aromaticity, showing negative values in cases where other indicators and bond distances suggest non-aromaticity. This is particularly true for rings fused with highly aromatic systems with highly negative NICS values (−25 to −30 ppm), although it can also happen with lower values. Thus, relying solely on NICS can be misleading, and other computational indicators, as well as experimental or structural data, should be considered.

## Computational Details

All calculations were performed with the Gaussian 16 package [64] by means of the B3LYP [65–67] hybrid density functional and the 6-311++G(d,p) basis set [68]. The geometry optimizations were carried out without symmetry constraints (Table S2 in Supporting Information File 1). Analytical Hessians were computed to characterize the optimized structures as minima (zero imaginary frequencies). Aromaticity was first evaluated by means of the nucleus-independent chemical shift (NICS) [12,69–71], proposed by Schleyer and co-workers as a magnetic descriptor of aromaticity. NICS is defined as the negative value of the absolute shielding computed at a ring center or at some other point of the system. Rings with large negative NICS values are considered aromatic. NICS values were computed using the gauge-including atomic orbital method (GIAO) [72]. Multicenter indices (MCI) [54–56,73–74] were computed with the ESI-3D program using AIM partition of space [75–76]. The anisotropy of the induced current density (AICD) plots have been computed at the same level of theory [77]. Current density maps have been computed by means of the SYSMOIC package [78–80], at the same level of theory. Finally, the energy decomposition analysis has been performed at the ZORA-BLYP-D3(BJ)/TZ2P level of theory with AMS software [81–84].

## Supporting Information

File 1 Energy decomposition analysis of pyrazole^CC^ (fragments used, molecular orbitals overlaps, and fragment molecular orbitals), cartesian coordinates and energies of all compounds under analysis, and whole set of ring current density maps.

## Data Availability

All data that supports the findings of this study is available in the published article and/or the supporting information of this article.
