# Supplementary material for: Unraveling aromaticity: the dual worlds of pyrazole, pyrazoline, and 3D carborane
Source: Beilstein J Org Chem. 2025 Feb 21;21:412–20. doi: 10.3762/bjoc.21.29 (PMC11849550; doi:10.3762/bjoc.21.29)

BEILSTEIN JOURNAL OF ORGANIC CHEMISTRY

Supporting Information

for

Unraveling aromaticity: the dual worlds of pyrazole, pyrazoline, and 3D carborane

Zahra Noori, Miquel Solà, Clara Viñas, Francesc Teixidor and Jordi Poater

Beilstein J. Org. Chem. **2025,** *21,* 412–420. doi:10.3762/bjoc.21.29

Energy decomposition analysis of pyrazole^{CC} (fragments used, molecular orbitals overlaps, and fragment molecular orbitals), cartesian coordinates and energies of all compounds under analysis, and whole set of ring current density maps

License and Terms: This is a supporting information file under the terms of the Creative Commons Attribution License (https://creativecommons.org/licenses/by/4.0). Please note that the reuse, redistribution and reproduction in particular requires that the author(s) and source are credited and that individual graphics may be subject to special legal provisions.
The license is subject to the *Beilstein Journal of Organic Chemistry* terms and conditions: (https://www.beilstein-journals.org/bjoc/terms)

Table of contents

Figure S1: Model system used in the energy decomposition analysis of pyrazole^{CC}.

Table S1: Overlap matrix between the fragment molecular orbitals (FMOs) of the two fragments considered in the EDA analysis of pyrazole^{CC}. FMOs energies (in eV) also enclosed.

Figure S2: FMOs of each fragment considered in the EDA of pyrazole^{CC}.

Table S2: Cartesian coordinates (in Å) and electronic energies (in Hartrees) of all systems under analysis. Computed at B3LYP/6-311++G(d,p) level of theory.

Figure S3. Current density maps (all-electron contributions) for a perpendicular magnetic field over a plane 1 a.u. above the molecular plane of *o*-carboranes-fused pyrazole systems and reference systems. Red/blue arrows when the component parallel/antiparallel to **B** is greater than 30% of the vector modulus (top). Bond current strengths for a magnetic field perpendicular to the molecular plane (bottom). Values aside each arrow represent the percentage relationship with respect to a reference current strength of 12 nA/T.

Diatropic/paratropic circulations are clockwise / anticlockwise.

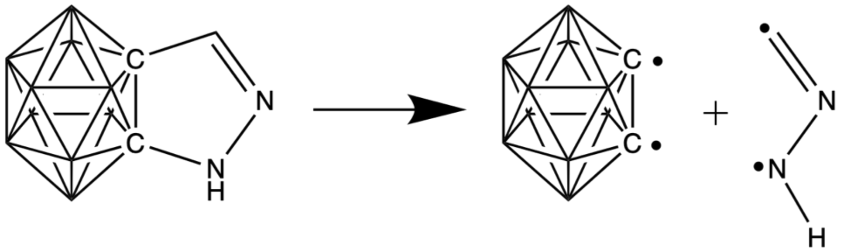

Figure S1: Model system used in the energy decomposition analysis of pyrazole^{CC}.

Table S1: Overlap matrix between the fragment molecular orbitals (FMOs) of the two fragments considered in the EDA analysis of pyrazole^{CC}. FMOs energies (in eV) also enclosed.

								**carboryne$^{\alpha\alpha}$**
		-8.42	-8.27	-8.18	-8.18	-6.91	-5.59	-1.35
**$N_2CH_2^{\beta\beta}$**		HOMO-4	HOMO-3	HOMO-2	HOMO-1	SOMO	SOMO'	LUMO
-11.91	HOMO-4	0.002	0.004	0.001	0.011	0.107	0.156	0.013
-11.41	HOMO-3	0.001	0.001	0.005	0.009	0.187	0.085	0.008
-10.02	HOMO-2	0.004	0.003	0.003	0.042	0.039	0.082	0.006
-7.21	SOMO	0.027	0.015	0.007	0.002	0.072	0.331	0.062
-5.25	HOMO-1	0.022	0.028	0.000	0.001	0.019	0.215	0.097
-4.94	SOMO'	0.018	0.009	0.009	0.004	0.255	0.158	0.022
-1.71	LUMO	0.001	0.017	0.014	0.047	0.022	0.005	0.036

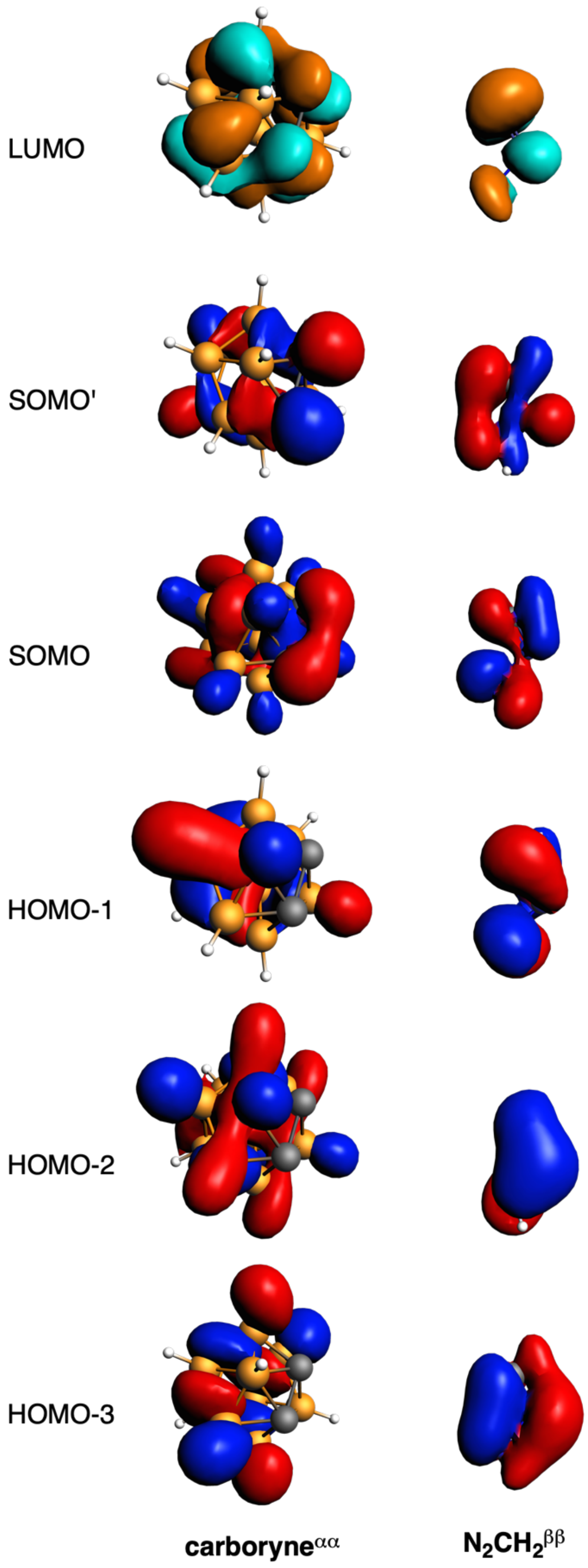

Figure S2: FMOs of each fragment considered in the EDA of pyrazole^{CC}.

Table S2: Cartesian coordinates (in Å) and electronic energies (in Hartrees) of all systems under analysis. Computed at B3LYP/6-311++G(d,p) level of theory.

Indazole (-379.9767278)

1	7	0	-2.264576	0.835361	0.000000
2	7	0	-1.099799	1.535108	0.000000
3	6	0	1.745971	-1.418451	0.000000
4	6	0	0.388873	-1.684127	0.000000
5	6	0	-0.505526	-0.600754	0.000000
6	6	0	0.000000	0.722724	0.000000
7	6	0	1.374234	0.996285	0.000000
8	1	0	-2.692626	-1.198629	0.000000
9	1	0	0.021871	-2.703609	0.000000
10	6	0	2.230422	-0.090891	0.000000
11	6	0	-1.925986	-0.438763	0.000000
12	1	0	2.455806	-2.236733	0.000000
13	1	0	1.753127	2.011119	0.000000
14	1	0	3.300815	0.078181	0.000000
15	1	0	-1.136295	2.540244	0.000000

Indazoline (-381.161345)

1	7	0	-2.398264	0.084525	0.126066
2	7	0	-1.490581	1.209274	-0.011420
3	6	0	2.218541	-0.712610	0.034381
4	6	0	1.011243	-1.419036	0.010931
5	6	0	-0.179294	-0.709648	-0.016715
6	6	0	-0.170547	0.686482	-0.016043
7	6	0	1.020474	1.401982	0.000613
8	1	0	-1.718404	1.713474	-0.862326
9	1	0	1.012399	-2.503481	0.004546
10	6	0	2.217887	0.682522	0.020820
11	6	0	-1.618724	-1.154809	-0.133804
12	1	0	3.158232	-1.250870	0.062241
13	1	0	1.019105	2.485332	0.009988
14	1	0	3.159506	1.218807	0.040562
15	1	0	-1.906276	-1.929273	0.580246
16	1	0	-1.829595	-1.531994	-1.142238
17	1	0	-2.670528	0.092114	1.103372

Pyrazole (-226.280132)

1	6	0	-0.745660	-0.892221	0.000000
2	6	0	0.664322	-0.997500	0.000000
3	6	0	1.111723	0.308120	0.000000
4	7	0	0.000000	1.087278	0.000000
5	7	0	-1.145889	0.376077	0.000000
6	1	0	1.265179	-1.891315	0.000000
7	1	0	-0.050690	2.092728	0.000000
8	1	0	-1.480068	-1.682961	0.000000
9	1	0	2.104488	0.727665	0.000000

Pyrazoline (-227.4485913)

1	6	0	1.008512	0.670997	0.044739
2	6	0	-0.206060	1.218056	0.055058
3	6	0	-1.226560	0.114542	-0.117672
4	7	0	-0.423396	-1.130778	0.030319
5	7	0	0.979341	-0.744811	0.005213
6	1	0	-0.433751	2.271038	0.123905
7	1	0	-0.568366	-1.494094	0.965865
8	1	0	1.970094	1.165186	0.078950
9	1	0	-1.703847	0.161981	-1.106605

10	1	0	1.413205	-1.122254	-0.833436
11	1	0	-2.024302	0.125693	0.629854

Pyrazonline2 (-227.4692596)

1	6	0	1.187559	-0.199182	-0.080545
2	6	0	0.492284	1.131843	0.108132
3	6	0	-0.962229	0.698607	-0.140396
4	7	0	-0.900494	-0.736728	0.212624
5	7	0	0.393912	-1.200405	-0.033077
6	1	0	0.637703	1.491638	1.134533
7	1	0	-1.574103	-1.335855	-0.246228
8	1	0	2.254099	-0.351786	-0.181897
9	1	0	-1.228729	0.839037	-1.197319
10	1	0	-1.688091	1.219205	0.483481
11	1	0	0.839505	1.910082	-0.572545

Pyrazole^{CC} (-479.8371731)

1	5	0	1.553756	0.011744	1.458400
2	1	0	2.131376	0.025900	2.487100
3	5	0	2.078353	0.922695	0.017105
4	1	0	3.056569	1.582171	0.030554
5	5	0	0.622595	1.437498	0.896889
6	1	0	0.432704	2.449640	1.470542
7	6	0	-0.646572	0.776310	-0.017432
8	7	0	-2.038532	1.077644	-0.084428
9	5	0	2.116155	-0.874559	0.020892
10	1	0	3.121325	-1.491946	0.033177
11	5	0	0.701190	-1.445641	-0.891480
12	1	0	0.565754	-2.461825	-1.471421
13	5	0	1.592084	0.008309	-1.431992
14	1	0	2.196837	0.015908	-2.444866
15	5	0	0.651799	1.436892	-0.902498
16	1	0	0.477270	2.448510	-1.479471
17	5	0	0.686432	-1.447385	0.901043
18	1	0	0.537898	-2.464764	1.475996
19	5	0	-0.177729	-0.023447	1.475647
20	1	0	-0.994542	-0.032423	2.319937
21	5	0	-0.142835	-0.019799	-1.492623
22	1	0	-0.939525	-0.028385	-2.354614
23	6	0	-0.622675	-0.828470	-0.014900
24	1	0	-2.404016	1.877775	0.411069
25	1	0	-2.517583	-2.100224	0.008885
26	6	0	-2.081036	-1.113409	-0.006165
27	7	0	-2.814520	-0.057424	0.011166

Pyrazole^{CB} (-479.8456006)

1	5	0	1.580787	0.043351	-1.434532
2	1	0	2.119793	-0.100947	-2.471607
3	5	0	2.047150	-0.952921	-0.037616
4	1	0	2.916651	-1.740215	-0.139505
5	6	0	0.604957	-1.239815	-0.863189
6	1	0	0.437964	-2.144629	-1.427932
7	6	0	-0.671310	-0.687400	0.019358
8	7	0	-2.017121	-1.091669	-0.119374
9	5	0	2.170651	0.831308	0.033038
10	1	0	3.225285	1.360016	0.036535
11	5	0	0.775345	1.446445	0.955072
12	1	0	0.825251	2.424578	1.613613
13	5	0	1.584730	-0.083921	1.446628
14	1	0	2.207372	-0.200809	2.441724

15	5	0	0.588700	-1.437919	0.862178
16	1	0	0.409037	-2.520132	1.288187
17	5	0	0.758554	1.508571	-0.838631
18	1	0	0.789362	2.517519	-1.449591
19	5	0	-0.169677	0.151368	-1.439098
20	1	0	-0.884747	0.036122	-2.362536
21	5	0	-0.159856	0.045319	1.477933
22	1	0	-0.897984	-0.056761	2.389969
23	5	0	-0.696652	0.986032	0.050453
24	1	0	-2.338832	-1.885416	0.417338
25	1	0	-2.878585	2.020432	0.064838
26	6	0	-2.261593	1.130310	0.031999
27	7	0	-2.891671	0.003599	-0.010220

Pyrazole^{BB} (-479.8762496)

1	5	0	1.558536	0.009623	1.451045
2	1	0	2.353079	0.030217	2.317513
3	6	0	1.940649	0.849211	-0.000705
4	1	0	2.900953	1.339886	-0.001092
5	5	0	0.639060	1.442229	0.902650
6	1	0	0.790528	2.453469	1.488197
7	5	0	-0.770952	0.844623	0.000427
8	7	0	-2.210453	1.089577	-0.000192
9	6	0	1.983007	-0.796985	-0.000210
10	1	0	2.966004	-1.240425	-0.000326
11	5	0	0.701889	-1.454799	-0.898714
12	1	0	0.899743	-2.458233	-1.483454
13	5	0	1.557729	0.008711	-1.451630
14	1	0	2.351510	0.028654	-2.318811
15	5	0	0.638562	1.441638	-0.903422
16	1	0	0.789013	2.452838	-1.489299
17	5	0	0.702455	-1.454351	0.899399
18	1	0	0.900838	-2.457290	1.484806
19	5	0	-0.170958	-0.028169	1.463305
20	1	0	-0.801428	-0.034516	2.459516
21	5	0	-0.171757	-0.028997	-1.462617
22	1	0	-0.803350	-0.036056	-2.458102
23	5	0	-0.737987	-0.915126	0.000785
24	1	0	-2.741779	1.941945	0.000209
25	1	0	-2.856455	-2.066835	0.000440
26	6	0	-2.282080	-1.147908	0.000435
27	7	0	-2.979691	-0.047643	-0.000216

Pyrazolyne^{CC} (-481.0461566)

1	5	0	1.526498	0.042035	1.517302
2	1	0	2.043976	0.077901	2.577260
3	5	0	2.146954	0.902951	0.082655
4	1	0	3.132713	1.550154	0.129051
5	5	0	0.653820	1.462091	0.858386
6	1	0	0.442788	2.490103	1.396109
7	6	0	-0.580682	0.792166	-0.110666
8	7	0	-1.966796	1.158442	-0.296254
9	5	0	2.161206	-0.889482	0.139128
10	1	0	3.154486	-1.520279	0.228104
11	5	0	0.789492	-1.471132	-0.832884
12	1	0	0.669924	-2.501563	-1.392097
13	5	0	1.728569	-0.046655	-1.367216
14	1	0	2.394423	-0.073986	-2.340854
15	5	0	0.774559	1.408831	-0.928288
16	1	0	0.651800	2.402967	-1.547388

17	5	0	0.668628	-1.417536	0.945080
18	1	0	0.464650	-2.412434	1.543183
19	5	0	-0.204043	0.031105	1.413179
20	1	0	-1.043001	0.053513	2.238626
21	5	0	0.000502	-0.057918	-1.515987
22	1	0	-0.736504	-0.079421	-2.428470
23	6	0	-0.576145	-0.808350	-0.058098
24	1	0	-2.218165	2.012698	0.187306
25	7	0	-2.799533	0.066281	0.186866
26	6	0	-2.068629	-1.164577	-0.156435
27	1	0	-2.346994	-1.954925	0.538342
28	1	0	-2.304402	-1.471597	-1.174519
29	1	0	-2.819583	0.116927	1.205478

Pyrazolyne^{CB} (-481.0603202)

1	5	0	1.557394	0.040643	-1.488313
2	1	0	2.040871	-0.097494	-2.553363
3	5	0	2.133110	-0.913851	-0.105408
4	1	0	3.017614	-1.679528	-0.239313
5	6	0	0.658956	-1.253135	-0.847765
6	1	0	0.472985	-2.167485	-1.390108
7	6	0	-0.598096	-0.707059	0.085677
8	7	0	-1.921157	-1.236222	0.048565
9	5	0	2.208865	0.868151	-0.065721
10	1	0	3.247784	1.424408	-0.125253
11	5	0	0.850740	1.462232	0.919787
12	1	0	0.911255	2.447322	1.567868
13	5	0	1.718719	-0.043245	1.390654
14	1	0	2.396722	-0.134443	2.351852
15	5	0	0.725633	-1.425245	0.863159
16	1	0	0.592004	-2.502683	1.316129
17	5	0	0.732697	1.499538	-0.865280
18	1	0	0.710888	2.497649	-1.494927
19	5	0	-0.194908	0.107461	-1.384297
20	1	0	-0.943169	-0.031335	-2.280374
21	5	0	-0.026114	0.047179	1.497947
22	1	0	-0.717443	-0.063554	2.447196
23	5	0	-0.660120	0.967276	0.109662
24	1	0	-2.142254	-1.664892	0.940449
25	1	0	-3.065869	-0.168275	-1.154950
26	6	0	-2.244529	1.186392	0.170002
27	7	0	-2.857296	-0.146683	-0.163730
28	1	0	-2.621809	1.914487	-0.549887
29	1	0	-2.578470	1.498270	1.162402

Pyrazolyne^{BB} (-481.0796582)

1	5	0	1.552997	-0.022693	1.494265
2	1	0	2.303613	-0.031212	2.399306
3	6	0	2.015839	0.821748	0.077585
4	1	0	2.983107	1.297060	0.122501
5	5	0	0.685418	1.431364	0.922138
6	1	0	0.824139	2.430203	1.530528
7	5	0	-0.706942	0.866897	-0.050415
8	7	0	-2.126818	1.257768	-0.062647
9	6	0	2.032343	-0.815506	0.055151
10	1	0	3.007053	-1.275609	0.094658
11	5	0	0.788188	-1.443434	-0.906181
12	1	0	0.995169	-2.442721	-1.495839
13	5	0	1.684380	0.015026	-1.407776
14	1	0	2.523070	0.037719	-2.231836

15	5	0	0.756835	1.452865	-0.868119
16	1	0	0.950700	2.464338	-1.441112
17	5	0	0.691487	-1.464085	0.880686
18	1	0	0.843128	-2.473719	1.469129
19	5	0	-0.183736	-0.025113	1.415814
20	1	0	-0.855747	-0.040969	2.386669
21	5	0	-0.047160	-0.002405	-1.487442
22	1	0	-0.631717	0.011137	-2.513531
23	5	0	-0.684534	-0.892004	-0.074629
24	1	0	-2.450538	1.725747	-0.899009
25	7	0	-2.964735	0.080040	0.143335
26	6	0	-2.249488	-1.210039	-0.153824
27	1	0	-2.598456	-1.954177	0.566246
28	1	0	-2.540445	-1.560225	-1.149266
29	1	0	-3.189038	0.088464	1.131571

Figure S3. Current density maps (all-electron contributions) for a perpendicular magnetic field over a plane 1 a.u. above the molecular plane of *o*-carboranes-fused pyrazole systems and reference systems. Red/blue arrows when the component parallel/antiparallel to **B** is greater than 30% of the vector modulus (top). Bond current strengths for a magnetic field perpendicular to the molecular plane (bottom). Values aside each arrow represent the percentage relationship with respect to a reference current Diatropic/paratropic circulations are clockwise / anticlockwise.

Current density maps

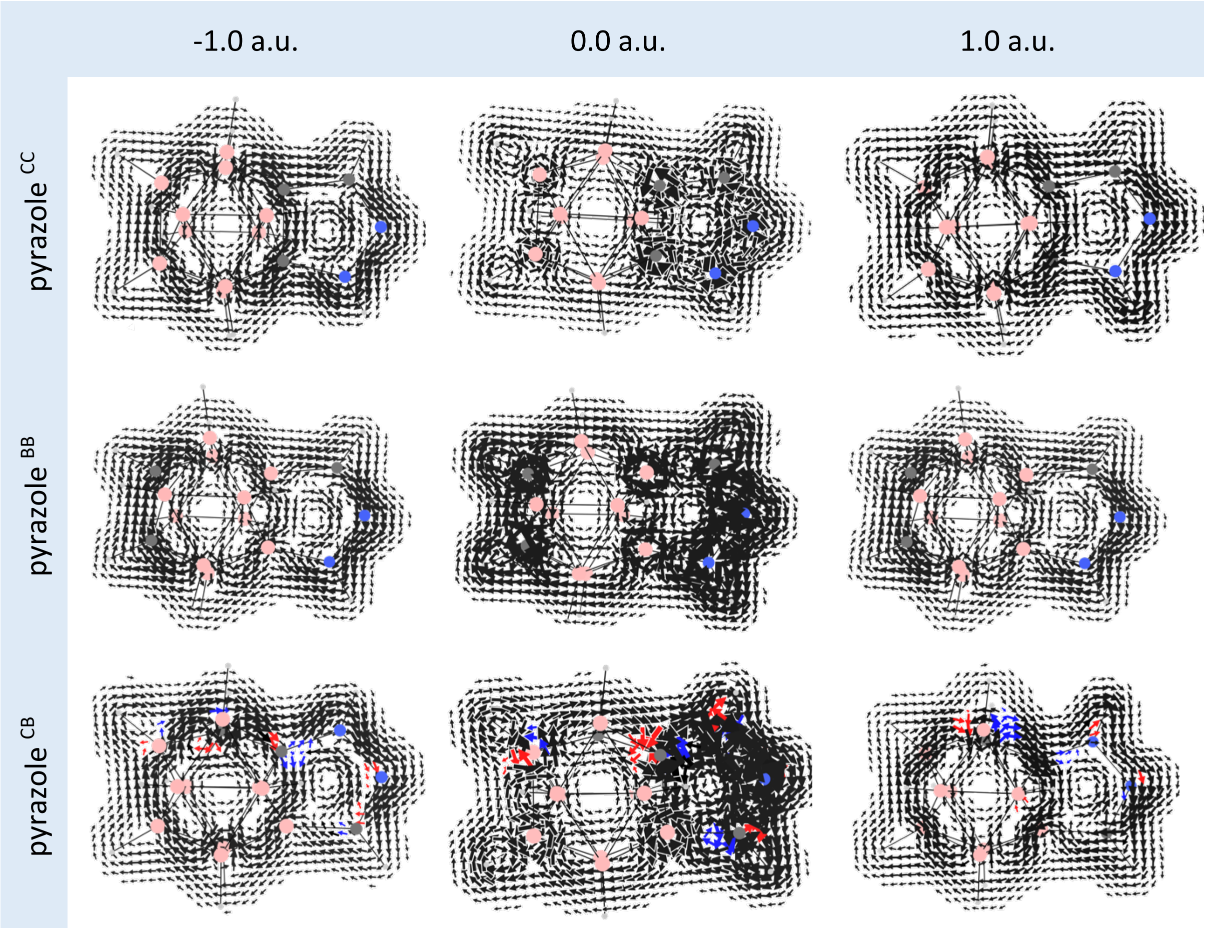

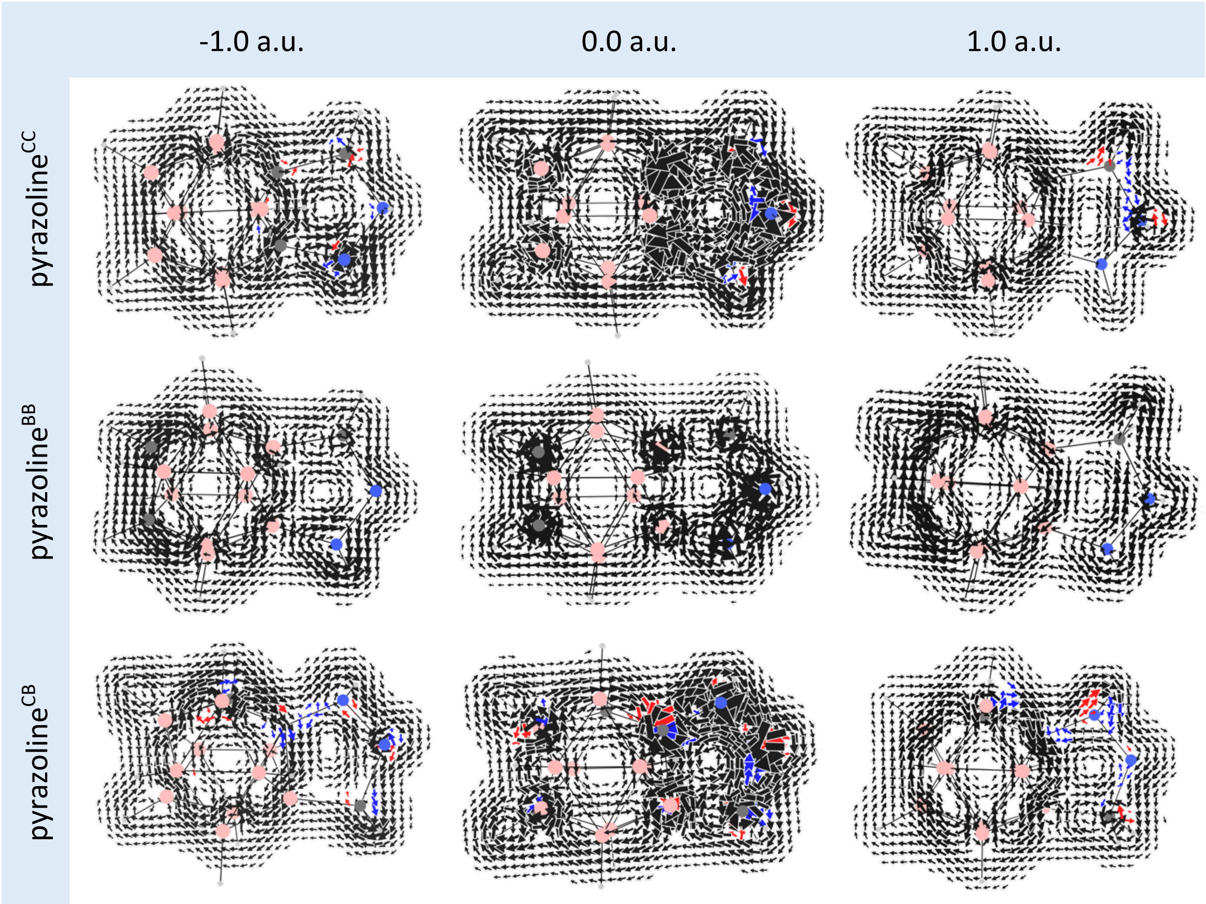

-1.0 a.u.
0.0 a.u.
1.0 a.u.
pyrazoline^{CC}
pyrazoline^{BB}
pyrazoline^{CB}

indazoline	indazole

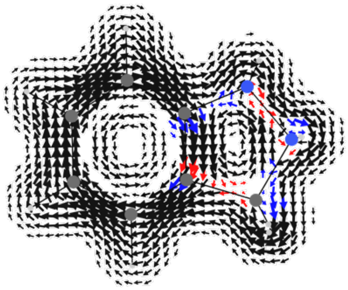

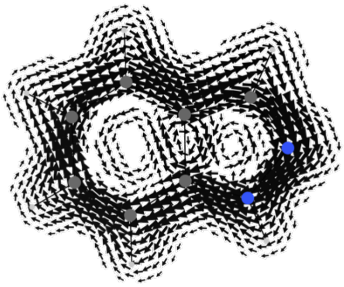

3-pyrazoline	2-pyrazoline

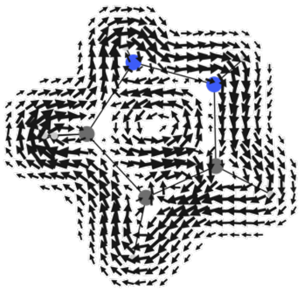

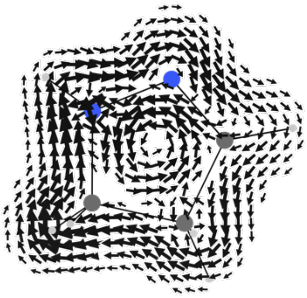

pyrazole

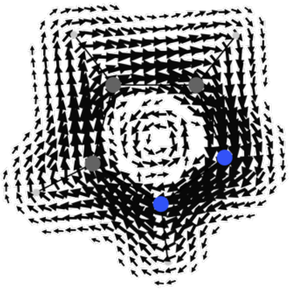

Bond current strengths

pyrazole^{CC}

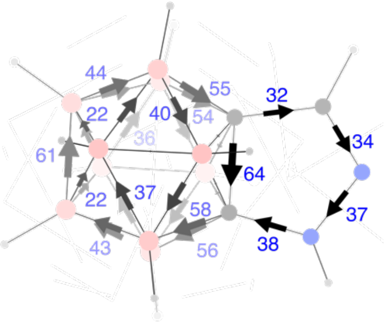

pyrazole^{BB}

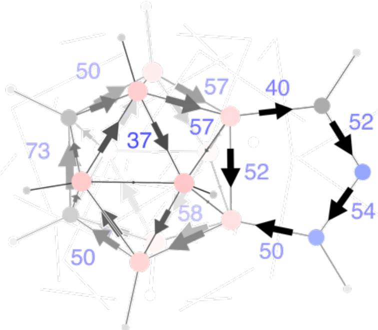

pyrazole^{CB}

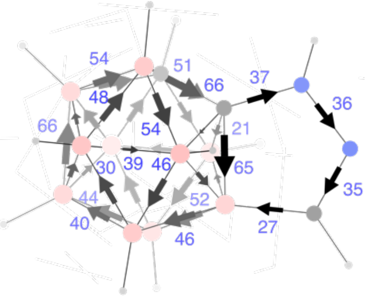

pyrazoline^{CC}

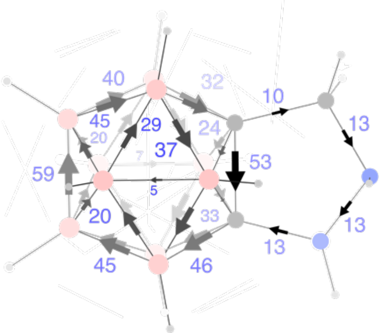

pyrazoline^{BB}

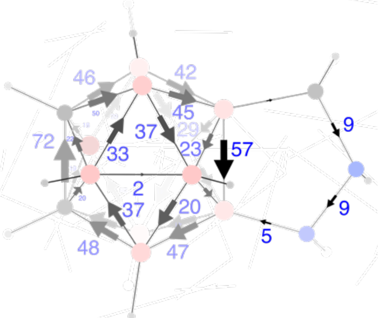

pyrazoline^{CB}

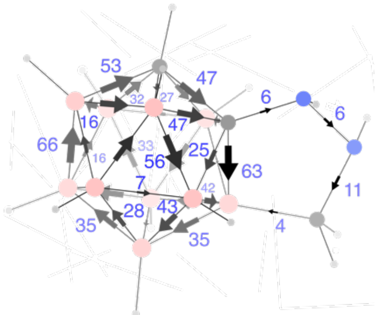

indazoline

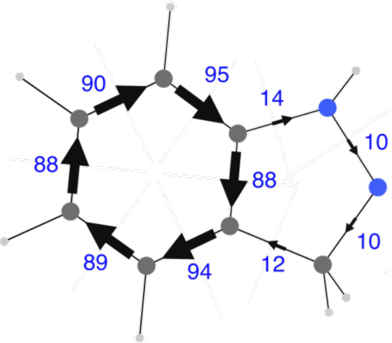

indazole

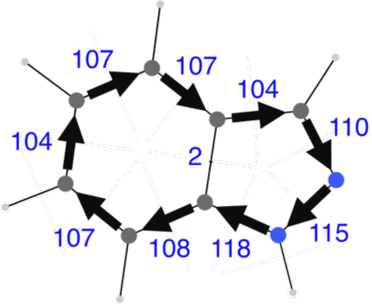

pyrazole

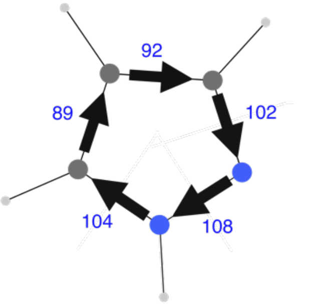

3-pyrazoline

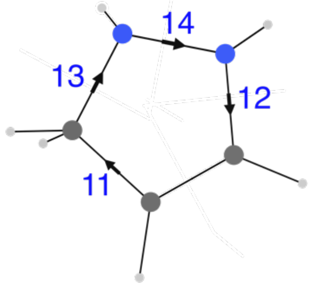

2-pyrazoline

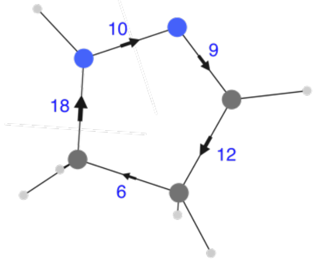

Supplement: File 1 — Energy decomposition analysis of pyrazoleCC (fragments used, molecular orbitals overlaps, and fragment molecular orbitals), cartesian coordinates and energies of all compounds under analysis, and whole set of ring current density maps. [file Beilstein_J_Org_Chem-21-412-s001.pdf]
